# The Wae to repair: prostaglandin E2 (PGE_2_) triggers intestinal wound repair

**DOI:** 10.15252/embj.201695973

**Published:** 2017-01-04

**Authors:** Rene Jackstadt, Owen James Sansom

**Affiliations:** ^1^ CRUK Beatson InstituteUniversity of GlasgowGlasgowUK

**Keywords:** Development & Differentiation, Molecular Biology of Disease, Stem Cells

## Abstract

Accurate wound repair is a crucial step to protect organisms from environmental damage, for example infection and toxin exposure. In this issue of *The EMBO Journal*, Miyoshi *et al* (2017) have elucidated a new mechanism underpinning this process within the intestine where mesenchymal prostaglandin E2 produced following damage drives intestinal regeneration.

The intestine requires an efficiently orchestrated repair process given the contents of the intestinal lumen and its primary function as an absorptive organ. Unless damage is repaired quickly, barrier function would be compromised which would rapidly lead to infection, sepsis and ultimately death. After injury, the highly replicative nature of the intestine leads to rapid wound closure, to minimize the exposure to luminal antigens and microorganisms (Peterson & Artis, 2014). However, given repair/wound healing often activates many pathways deregulated in cancer, for example WNT and TGFβ, uncontrolled repair and loss of homoeostasis could lead to carcinogenesis (Ashton *et al*, 2010). To guarantee proper wound repair, a process termed epithelial restitution occurs in the intestine to rapidly reseal superficial injuries (Lacy, 1988). The healing process is most likely to be performed by a cell population that has transient repair features, wound‐associated epithelial (WAE) cells, and the induction of non‐canonical Wnt signalling is required (Stappenbeck & Miyoshi, 2009). WAE cells are controlled by an orchestra of growth factors and chemokines. However, both upstream activators of WAE cells and downstream effector pathways need to be elucidated.

**Figure 1:**
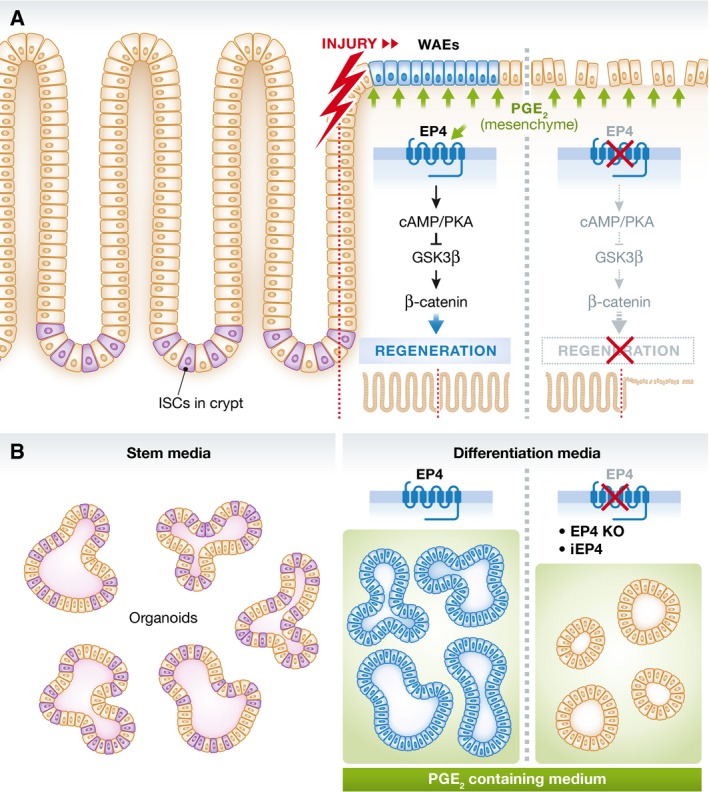
Mechanistic model describing the role of PGE_2_–EP4–PKA–ß‐catenin signalling in intestinal wound repair (A) During intestinal injury, PGE_2_ levels increase locally. This triggers a non‐canonical Wnt signalling cascade resulting in regeneration and wound repair. Pharmacological inhibition or genetic deletion of EP4 impairs this signalling and the tissue remains refractory for regeneration and wound healing. (B) *In vitro* organoid propagation using stem cell medium enables the culture of stem cell rich organoids. Growth factor‐depleted but PGE_2_‐containing medium leads to WAE cell differentiation if cells are EP4 proficient; those cells resemble their *in vivo* analogue. EP4 deficiency or pharmacological inhibition of EP4 (iEP4) leads to enterocyte differentiation and a reduced regenerative capacity.

In an elegant study, Miyoshi *et al* (2017) used a series of models to address this important question: mouse, organoid and human cell lines. Transcriptomic profiling revealed that postmitotic properties of WAE cells can be generated by growth factor depletion of intestinal organoids. This resembles an atypical differentiation state of WAE cells that has been previously described during the process of epithelial reconstitution (Lacy, 1988; Stappenbeck & Miyoshi, 2009). Importantly, this differentiation state was prostaglandin E2 (PGE_2_) dependent. Using a genetically engineered model for *Ptger4* (encoding for the prostaglandin E receptor 4, EP4) deletion, the authors show that PGE_2_ triggered WAE cell differentiation is transmitted by EP4 *in vitro* and *in vivo*. In an array of elegant *in vitro* assays, the authors could show that the PGE_2_–EP4 axis irreversibly leads to WAE cell differentiation, while inhibition of the PGE_2_–EP4 axis leads to enterocyte differentiation. Mechanistically, the authors describe that EP4 signalling acts via cAMP–PKA–GSK3β cascade that controls β‐catenin stabilization (Wnt signalling activity) and eventually differentiation to WAE cells. Notably, the authors demonstrated a concentration‐dependent differentiation into WAE cells, which may explain the spatial–temporal appearance of WAE cells during healing. To note, mesenchymal cells as source for PGE_2_ have been described by the authors previously (Manieri *et al*, 2015).

Cancer has been suggested to be a “wound that cannot heal” so it is notable that many of the pathways described here have been suggested to be important in colorectal carcinogenesis. Stromal COX2 has been shown to be important for growth of adenomas in *Apc* ^+/−^ mice, and COX2 produces Prostaglandin H2 of which Prostaglandin E2 is a downstream metabolite. Loss of EP2 and EP4 was shown to suppress tumorigenesis in *Apc* ^+/−^ mice (Sonoshita *et al*, 2001, 2002) and, recently, a PGE_2_–EP4 axis was suggested to control colon cancer stem cell expansion and metastasis (Wang *et al*, 2015).

Taken together, this study using a sophisticated *in vitro* tool to study wound repair has generated important new findings into this process. This *in vitro* system may also be able to define many more novel regulators of wound repair. Importantly, trials have already been performed using EP4 agonist in inflammatory bowel disease (Nakase *et al*, 2010). It would be interesting to assess whether these patients had more WAE cells and whether they showed increased wound repair. Conversely, further work examining the inhibition of the Prostaglandin E2–EP4 axis in both the initiation and progression of colorectal cancer should provide key insights into the hijacking of repair processes by cancer cells.
